# Senolytics improve muscle adaptation in old mice

**DOI:** 10.18632/aging.204578

**Published:** 2023-03-06

**Authors:** Cory M. Dungan, Charlotte A. Peterson

**Affiliations:** 1 Department of Health, Human Performance, and Recreation, Baylor University, Waco, TX 76706, USA; 2 College of Health Sciences, University of Kentucky, KY 40536, USA; 3 Center for Muscle Biology, University of Kentucky, KY 40536, USA; 4 Department of Physical Therapy, University of Kentucky, KY 40536, USA

**Keywords:** senescence, skeletal muscle, aging, senolytics

Deletion of senescent cells improves numerous metabolic and physiologic conditions, including cardiac and renal function, fasting blood glucose, and physical function, to improve the overall lifespan and healthspan of aging organisms [1–3]. Genetic mouse models and senolytics, compounds that selectively kill senescent cells, have provided us with a deeper understanding of the maladaptive nature of senescent cells and the therapeutic benefit of their deletion, while also highlighting their necessity in certain situations [4]. However, to date, the contribution of senescent cells to aging in skeletal muscle is equivocal. Recently, our lab and others have utilized senolytics to examine the contribution of senescent cells to impaired muscle adaptability with age, including regeneration following injury and the anabolic response to mechanical overload, as well as any potential role in sarcopenia.

There is little evidence that senescent cells are present in aged muscle causing sarcopenia, but they appear to contribute to the impaired ability of muscle to adapt to exogenous stimuli. Work from our lab shows that seven days following BaCl_2_ muscle injury, there are approximately 250 times more senescence-associated β-galactosidase positive (SA β-Gal+) cells in injured muscle compared to uninjured in both young and old mice [1]. In young mice, these numbers return close to baseline after 28 days, however, the senescent cell burden remains elevated in old mice [1]. Treating mice with a senolytic cocktail of dasatinib and quercetin (D+Q) lowers the senescence burden in old mice [2], while subsequently reducing the inflammatory profile of the muscle and improving the regenerative response [1]. Using the fluorescent substrate, C_12_FDG, to isolate β-Gal+ cells by FACS, we demonstrate that the majority of β-Gal+ cells 7 days-post BaCl_2_ injury are CD11b+ and are likely macrophages at this early stage as they are not affected by D+Q. β-Gal+ cells appear to transition to senescence in muscle from old mice, developing a senescence-associated secretory phenotype (SASP) relative to cells isolated from young mice 14 days post injury [1]. Some of these findings were recently confirmed by the Muñoz-Cánoves lab [5], who show a greater abundance of senescent cells early in the regenerative process that is reduced over time and greater in old versus young mice. They also show a reduction in senescent cell abundance in response to D+Q treatment following injury, associated with improved regeneration and improvements in muscle force production. Using a senescent cell isolation approach comparable to ours, they also performed a comprehensive characterization of the SASP during muscle aging and regeneration [5]. They confirm that most senescent cells in muscle are likely derived from myeloid cells, but they also detected smaller satellite cell and fibroadipogenic cell populations that may contribute to the senescent phenotype. lt is important to note that our work shows young mice treated with D+Q display an attenuated regenerative response [1], whereas the Muñoz-Cánoves lab shows an improvement in the regenerative response of young mice as early as 7 days post injury [5]. Considering senescence has been shown to be required for the full regenerative response in skeletal muscle [4], the large overlap in phenotype between β-Gal+ macrophages and bona fide senescent cells likely contributes to the confusion and warrants further investigation.

In a model of muscle hypertrophy, old mice display a blunted hypertrophic response relative to young mice, which is accompanied by a greater senescent cell burden [6]. Treatment with D+Q improves the hypertrophic response in old mice, in addition to lowering the abundance of senescent cells [6]. In this model, we did not observe any change in many of the SASP genes, although genes that are crucial for extracellular matrix reorganization, along with genes that negatively regulate myostatin, were elevated [6]. In summary, senolytics effectively lower the protracted senescent cell burden that accompanies a regenerative or hypertrophic stimulus in muscle from aged mice, resulting in increased muscle fiber size.

Although there is no clear consensus on the accumulation of senescent cells in skeletal muscle during aging [1, 6–8], systemic deletion of senescent cells improves physical function [1–3] and has been implicated in slowing sarcopenia [3]. This apparent disconnect between a lack of senescent cells in muscle and improvements in age-associated conditions could be due to various technical or biological reasons. First, the markers used to identify senescent cells to date are not specific and may not capture the senescent cell phenotype in muscle. The detailed transcriptomic characterization of senescent cells, including the SASP, in aged muscle [6], may help to resolve this issue. Alternatively, senescent cells in aged muscle may be localized, for example, to the neuromuscular junction. Treatment with senolytics would limit the age-mediated loss in motor neurons, along with the subsequent denervation and loss of muscle mass and strength. Senolytics may also affect other cells in muscle, as we have shown increased myogenic progenitor cell proliferation *in vitro* in response to D+Q [1]. Or perhaps the improvement in muscle function, regeneration and hypertrophy are not due to internal changes in the muscle but from systemic effects. Senolytic-mediated improvements in the function of other tissues may be beneficial to skeletal muscle.

It is becoming increasingly clear that deletion of senescent cells improves physical function during aging but our work points to a more complex role in skeletal muscle adaptation. Advances in technology to quantify senescent cell burden and phenotype are likely to promote a more efficacious use of senolytics to maintain muscle mass and strength with age.
